# Device response principles and the impact on energy resolution of epitaxial quantum dot scintillators with monolithic photodetector integration

**DOI:** 10.1038/s41598-024-74160-7

**Published:** 2024-10-02

**Authors:** Allan Minns, Tushar Mahajan, Vadim Tokranov, Michael Yakimov, Michael Hedges, Pavel Murat, Serge Oktyabrsky

**Affiliations:** 1 College of Nanotechnology, Science and Engineering, University at Albany, State University of New York, Albany, NY USA; 2 Fermi National Accelerator Laboratory, Batavia, IL USA

**Keywords:** Quantum dots, Optical materials and structures, Electronics, photonics and device physics

## Abstract

Epitaxial quantum dot (QD) scintillator crystals with picosecond-scale timing and high light yield have been created for medical imaging, high energy physics and national security applications. Monolithic photodetector (PD) integration enables the sensing of photons generated within the waveguiding crystal and allows a wide range of scintillator-photodetector coupling geometries. Until recently, these doubly novel devices have suffered from complex, high variance responses to monoenergetic sources which significantly reduces their precision and accuracy. The principles governing the overall device response have now been discerned and embodied by an expression derived within a geometrical optics framework which considers optical properties, surface roughness and photodetector coupling geometry. Response variation due to these factors was sufficiently reduced to obtain material-related energy resolution values of 2.4% with alpha particles. These findings place energy resolution alongside luminescence timescale, photon yield, and radiation hardness as outstanding properties of these engineered materials.

## A nanostructured semiconductor scintillator

Scintillators primarily convert particles of ionizing radiation to quantifiable pulses of light. For many applications including particle calorimetry and radiation monitoring, single-digit energy resolution is a requirement placed on this quantification^1,2^. Our previous research into the nanoscale design and growth of materials has provided comparatively fast and high-yield alternatives to traditional scintillators^3–6^. However, variance in the response of monolithically integrated scintillator-photodetector devices has been prohibitively high, with full width at half maximum (FWHM) values typically exceeding 10%. Evidence has indicated a response dependence on scintillator-PD dimensions and coupling geometry^5,6^. This work represents an effort to parameterize the device response principles and delineate geometric versus material contributions to energy resolution.

**Fig. 1:**
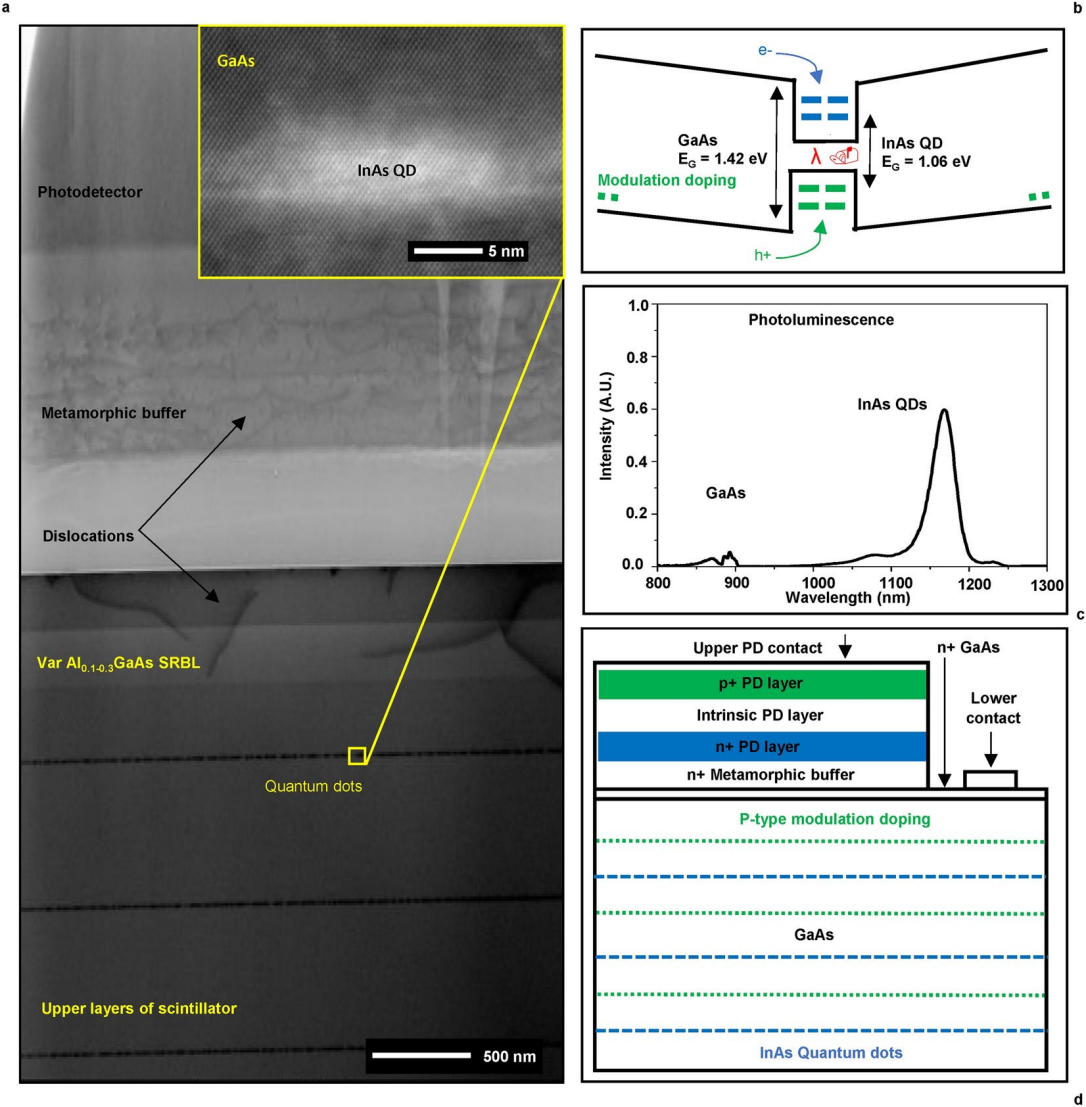
Scintillator crystal properties and overall device structure. (**a**) (Inset) High angle annular dark field scanning transmission electron microscopy (HAADF STEM) image of an individual quantum dot. (Main) Bright field (BF) STEM image showing the generic features. (**c**) Depiction of the heterostructured energy bands, states within and photon emission around a QD with modulation p-doping. (**d**) Photoluminescence based emission spectrum. Quantum confinement results in an InAs ground state peak at 1170 nm (1.06 eV) vs. 873 nm (1.42 eV) for GaAs. (**e**) Illustration the device structure for this work.

Molecular beam epitaxy (MBE) facilitates the wafer-scale growth of gallium arsenide layers bearing sheets of indium arsenide quantum dots (Fig. 1a, Supplementary Information Fig. S1). InAs, having a lattice constant 7% larger than that of GaAs, relaxes strain energy during deposition by agglomerating into pyramidal nanostructures (Fig. 1a). Due to the strain, the bandgap offsets of the conduction and valence bands in the InAs QDs are about − 0.24 and 0.18 eV respectively, consequently the carriers see the QDs as deep potential wells with strong confinement at room and elevated temperatures. The combination of strain and quantum confinement result in atom-like energy level discretization in QDs and an average ground state transition of 1.06 eV^4^ (Fig. 1b,c). These effects enable the QDs to take on the role of radiative recombination centers found in conventional scintillators. The QD sheets have a planar density on the order of 4*10^10^ QDs/cm^2^ and are capped with AlAs monolayers to preserve the shape and size distributions^7^. Variable Al_0.1–0.3_GaAs surface recombination barrier layers (SRBL) are incorporated at the top and bottom of the scintillator.

The regions between the QD layers contain a 10 nm layer of carbon-doped p-type GaAs flanked by 200 nm of intrinsic GaAs. This modulation doping creates a drift field toward the QDs and ensures the QD hole states remain ready for radiative recombination. Electrons are captured by the positively charged QDs within several ps due to this field and their low effective mass^4,8^. InAs QDs are direct bandgap materials with an average radiative recombination time of approximately 0.5 ns at room temperature^9^. These factors comprise the origin of the ultrafast (< 1 ns) scintillation response shown previously^4–6^. The number of electrons generated by an ionizing particle can be estimated as ${\text{N}}_{\text{e}-\text{h}}=\frac{{\text{E}}_{\text{d}}}{\upbeta {\text{E}}_{\text{g}}}$ where E_g_ is the material band gap energy, E_d_ is the energy deposited by the ionizing particle and β is a constant between 2 and 3^10^. The 1.42 eV band gap of GaAs is much lower than many inorganic scintillator materials^10^, allowing an extremely high potential light yield of 240,000 photons per MeV of deposited energy. Previously, we have assessed linearity of the scintillation response using alpha particles with energies ranging from 1 to 4 MeV in energy^6^. The results agreed with theoretical predictions of photon yield, based on an observed QD efficiency of 50% at room temperature and a calculated PD external quantum efficiency of 60%^4^. As light yield is inversely proportional to the statistical component of energy resolution, these materials potentially enable low variance sensing over a wide range of device efficiencies.

### Monolithic photodetector integration and characterization

**Fig. 2:**
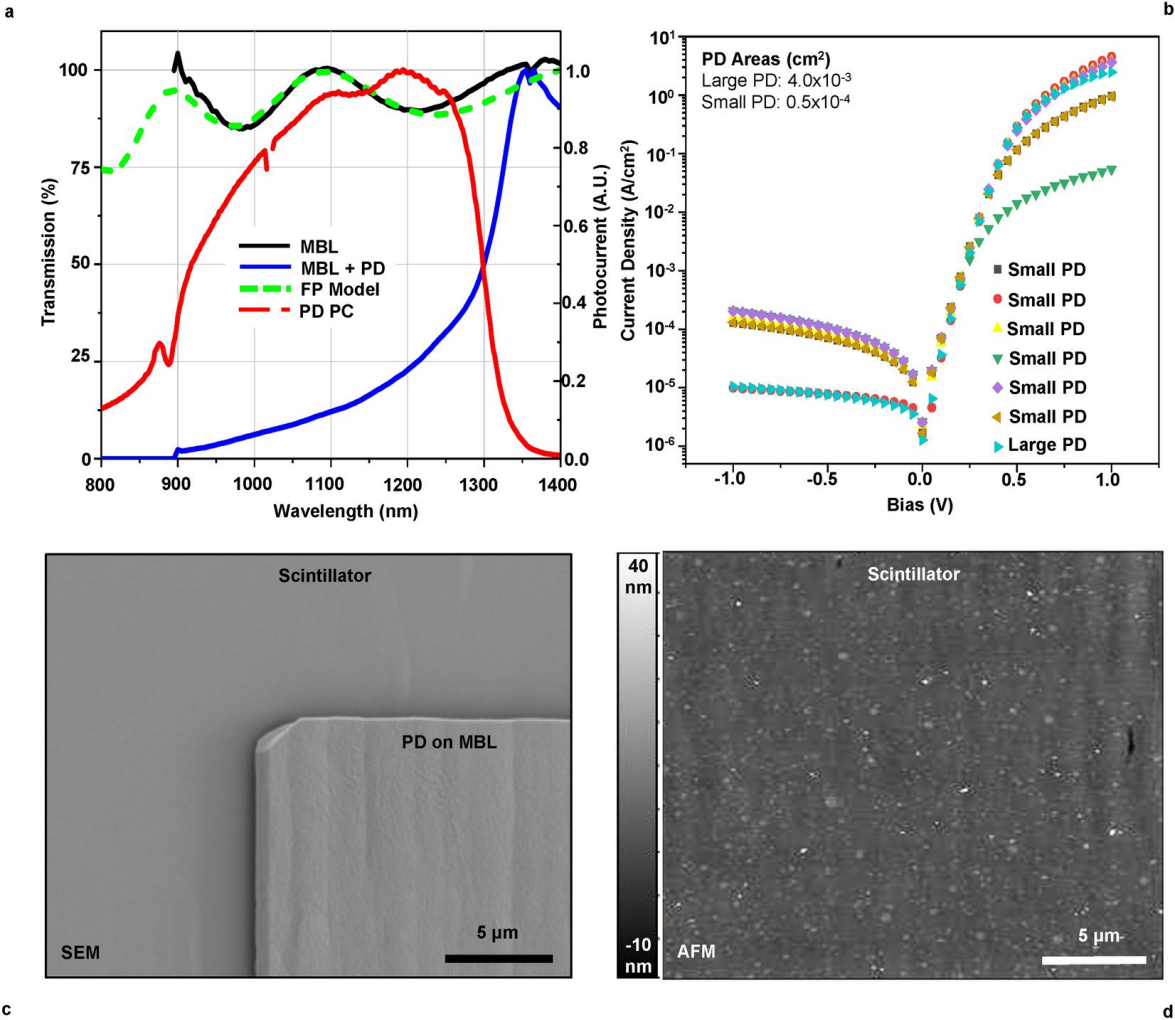
Characterizing the MBL, PD and surface qualities. (**a**) Optical transmission spectra of an MBL and MBL + PD on 350 μm thick GaAs wafers and PC spectrum from the PD. The fringes of the MBL layer are matched through a Fabry–Pérot (FP) interferometer response with III-V index models^11^ and a MBL thickness of 630 nm. MBL transparency abruptly tends toward zero under 900 nm. (**b**) Current–voltage curves taken from multiple PDs on a large sample of material. **c** SEM image of a PD/MBL on scintillator before contact metallization showing surface morphology from enhanced etching induced by strain fields around dislocations. (**d**) AFM data taken from a random sample of scintillator after contact metallization showing the etch driven surface morphology and contamination. RMS roughness of the scintillator surface is 2.3 nm.

Post growth, a sacrificial AlAs layer allows the substrate wafer to be released from the bottom of the 20–25 μm scintillator crystals using an epitaxial lift-off method^12^, revealing a pristine lower surface for exposure to ionizing radiation as shown in Fig. 1d. The high refractive index of GaAs versus air or adjacent substrate materials results in electromagnetic wave total internal reflection (TIR) and the confinement of photons within the scintillator. Monolithic photodetector integration offers a means of extracting light by incorporating the scintillator and PD into a contiguous material of homogenous permittivity. The PD with a 700 nm intrinsic In_0.35_Ga_0.65_As absorber layer is deposited during the final stages of epitaxial growth, has a p-i-n structure for low capacitance to support GHz bandwidth^4,5^ and is spectrally matched through the In content to the scintillator emission wavelengths. To accommodate the change in lattice constants between the scintillator and photodetector, an 800 nm variable Al_0.92–0.6_In_0.03–0.35_Ga_0.05_As metamorphic buffer layer (MBL) is incorporated during growth. The MBL has constant transparency across the QD emission range, as shown in Fig. 2a. The transmission spectrum with interference fringes was fitted with Airy distribution for Fabry–Pérot resonator using index models for III-V semiconductors. Given the complex composition of the MBL layer, the fit appears reliable as it has a scaling factor as the only fitting parameter. The observed fringe amplitude shows that GaAs/MBL interface introduces 0.5% reflection at normal incidence, which makes this interface good for optical coupling with the PD. The PD photocurrent (PC) response indicates maximum sensitivity at the characteristic QD ground state transition wavelength of ~ 1170 nm.

Strain from the 2.3% lattice mismatch between the scintillator and photodetector is released within the MBL as misfit dislocations^13^. Despite the presence of threading dislocations propagating into the photodetector as shown in Fig. 1a, the resultant current–voltage (I–V) curves given in Fig. 2b indicate manageable diode characteristics as the photodiode is operated in a zero-bias, photovoltaic mode. Dark current levels of the PD are at least an order of magnitude larger than similar commercial In_0.53_Ga_0.47_As photodetectors grown on a lattice-matched InP substrate. As the growth of the photodetectors sensitive to wavelengths > 1 μm on GaAs is uncommon, the results obtained from the metamorphic monolithic In_0.35_Ga_0.65_As photodetector can be considered successful.

### Origin of the response dependence on device geometry

**Fig. 3:**
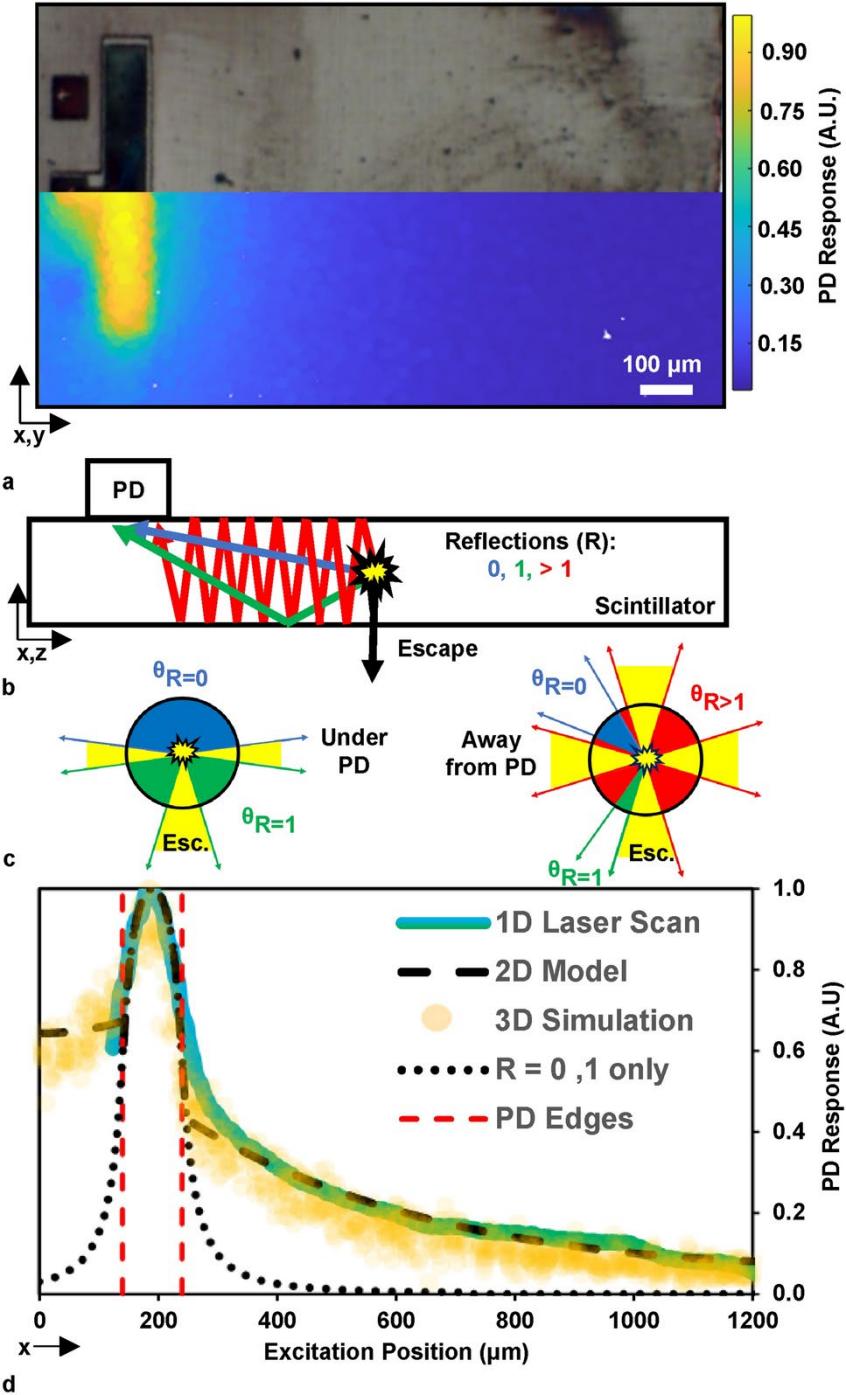
Photodetection on waveguiding scintillators. (**a**) Optical micrograph of a 100 μm wide photodetector covering a small portion of scintillator/waveguide overlaying color map results from a 3D Monte Carlo simulation of the same device. (**b**) Visualization of the R values for photon reflection before termination at the PD. (**c**) Partitioning the 2π plane angle about the averaged photon emission point into R values and escape wedges for two emission locations. (**d**) Laser scan, simulation, and model results for the device shown in (**A**). The simulation and model use a scattering parameter of $\upgamma$= 0.05 and absorption coefficient α = 0.8 cm^-1^.

The photodetector layers of the crystal are etched through the lithographically defined mask, leaving portions of the scintillator surface uncovered (Fig. 2c). The propagation of photons through the scintillating medium has been previously described in detail^5^, and we now use a simple method to account for scintillator-photodetector coupling geometry in a mathematical model of the integrated device response. By considering the photon emission from an ionizing particle moving through the crystal as isotropic and originating from a single point, we can partition the 4π solid angle around that point. Depending on the emission location and trajectory, photon propagation may terminate through absorption, escape, detection without any reflections (R = 0), detection after one reflection (R = 1), or detection after multiple reflections (R > 1) as depicted in Fig. 3b. Once absorption (Beer–Lambert Law) is incorporated into this process, the overall device response is reproduced in a ray-optics framework. With these considerations, the intensity equation for photon propagation inside the planar scintillator^5^ can be adapted into the expression:

1 $$\uptheta_{{{\text{PD}}}} = \uptheta_{{{\text{R}} = 0,1}} {\text{e}}^{{ - \upalpha {\text{x}}_{{{\text{R}} = 0,1}} }} + { }\uptheta_{{{\text{R}} > 1}} {\text{e}}^{{ - \upalpha {\text{x}}_{{{\text{R}} > 1}} }} \left( {1 - \upgamma } \right)^{{\text{B}}}$$

Here, $\uptheta$_PD_ is the total solid angle about the emission point consumed by photons which intersect the PD area, θ_R=0,1_ and X_R=0,1_ are the solid angle of the PD and propagation distances for photons with the number of reflections (R) equal to 0 and 1. θ_R>1_ is the solid angle outside the escape cones (2-dimensional rotation of a critical angle vector) or R = 0,1 angles. X_R>1_ are the forward and backward propagation distances for R > 1 photons. α is the absorption coefficient, $\upgamma$ is the scattering parameter and B is the number of reflections for the forward and backward waveguided rays. This method of partitioning can replace the ray tracing of individual photons and be used to estimate the fraction of emitted light incident upon the PD.

Previous observations on the use of a scattering parameter based on the Raleigh-Rice formula ${\upgamma } = {\text{e}}^{{\left( {\frac{4\uppi \upsigma \cos \uptheta }{\uplambda }} \right)^{2} }}$^5^, where σ is the RMS roughness, λ is the incident wavelength and θ is the angle of incidence, indicated a roughness 10 times greater than that observed with atomic force microscopy (AFM). Concordantly, recent roughness values (Fig. 2d) result in a scattering factor one order of magnitude lower than the device response indicates through modeling. While the conditions for the smooth surface approximation used by the Raleigh-Rice formula are met $\left( {\frac{{4\pi \upsigma {\text{cos}}\uptheta }}{\uplambda } < < 1} \right)$^14^, the total area sampled by AFM to quantify surface roughness is much less than 1%. Modeling the effects of scattering on the propagation of light is an active area of investigation, yet this simplified expression for $\upgamma$ enables a realistic device model albeit with a constant offset between calculated and observed values.

Figure 3a shows a comparison wherein a device was scanned with a laser beam, exposed to alpha particles in a Monte Carlo simulation and mathematically modeled as shown in supplementary information (SI) Sections 2 and 4. The waveguiding scintillator is over 1 mm in length and 25 μm thick, with a 100 μm wide PD on top. To simplify calculations, a 2D model with unit circle plane angle partitioning is used, as shown for two positions along the center of the scintillator in Fig. 3c. Waveguided light collection (R > 1) dominates in scintillator regions not covered by the PD. Approximately 88% (2% per surface) of emitted photons are TIR-confined to the scintillator. Losses due to absorption or re-direction into escape by scattering and photon recycling^15^ will further mitigate light collection by the PD. Therefore, exposure to a monoenergetic source may produce multimodal charge collection histograms with various distributions depending on the specific geometry of a device and the excitation location within the scintillator^5,6^.

Directly under the PD, the 2π of plane angle about the emission point is mostly consumed by the PD (R = 0,1) and losses are minimized by the escape cones and R > 1 areas being partially blocked by the PD. Figure 3d shows an area of maximum collection efficiency centered under the photodetector where $\frac{\text{d}{\uptheta }_{\text{PD}}}{\text{dx}}\approx 0$, i.e. there is no response variance from device geometry. In lieu of taking the 1st derivative of Eq. (1), iterative use of the device model reveals that for the device in Fig. 3 which has a 100 μm wide PD, there is an area 14 μm wide under the PD where collection efficiency varies by less than 1%. A 550 μm wide PD on a 25 μm thick scintillator would allow an area of equally minimal variation 169 μm wide (see SI Fig. S7) which is conducive for experimentation.

### Impact of variance mitigation on energy resolution

**Fig. 4:**
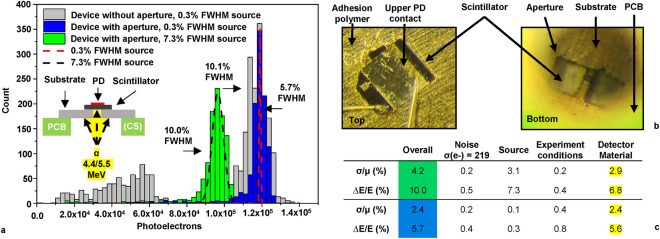
Experimental data and results. (**a**) Main: Bimodal and unimodal response of a single device in 3 experimental configurations in air. Energy resolution was improved with the use of targeted excitation. The 4.4 MeV source was 1.6–2.0 mm from the detector. The 5.5 MeV source surface was 4.4–13.4 mm from the detector (by angle) without an aperture and 4.4–5.4 mm from the detector with the aperture substrate. An overall device efficiency of 11% (26 e^−^/keV) was observed with both sources. Inset: Experimental diagram. (**b**) Top/bottom images of the device under test. (**c**) De-convolution of the energy resolution components.

A scintillation detector was prepared for exposure to low and high variance alpha particle sources with energy standard deviation/mean (σ/μ) values of 0.13% and 3.1%, as depicted in Fig. 4a. The I–V curves for this detector are given in Fig. 2b (Large PD). The 25 μm thick scintillator was cleaved down to a 500 × 600 μm specimen with 70% surface coverage by the PD. It was mounted on a 100 μm thick stainless steel substrate and partially aligned over the centroid of two adjacent 125 μm wide apertures as shown in Fig. 4b. Before substrate mounting the device was measured with a flood exposure from a 5.5 MeV source with σ/μ = 0.13%. After substrate mounting the device was re-exposed to the same source as well as a 4.4 MeV source with σ/μ = 3.1%. Due to the low source activities, scintillator thickness, overall detector efficiency and brief data collection durations, 60 keV gamma rays from Am-241, characteristic x-rays from the crystal and Compton scattered signals are not definitively observed.

The ability to discern the transition from 10.1% FWHM with flood exposure to 5.7% FWHM with targeted exposure and the same source is due to a reduction in the response variance from the detector material. Energy resolution (∆E/E = FWHM/μ = 2.36 σ/μ) can be shown to be a quadrature sum of multiple uncorrelated sources^16,17^. In this work the major sources are readout noise (σ_N_), the source (σ_S_), experimental conditions (σ_C_) and the detector material (σ_Ml_: intrinsic crystal resolution, statistical contribution and PD collection efficiency). Accordingly, ${\upsigma }_{\text{Overall}}=\sqrt{{\upsigma }_{\text{N}}^{2}+{{\upsigma }_{\text{S}}^{2}+\upsigma }_{\text{C}}^{2}+{\upsigma }_{\text{M}}^{2}}$. σ_C_ originates from the diameters of the sources and their separation from the detector in air, resulting in varying path lengths and attenuation of alpha particles striking the detector (SI Fig. S10). σ_N_ is primarily composed of the noise at the input of the CSA and shaping stages. Capacitance at the CSA input had a negligible effect on noise (SI Fig. S11) in this study.

Parsing the components of energy resolution as shown in Fig. 4c reveals that with a low variance source the dominant contributor to peak broadening is the detector material, with the lowest σ/μ value obtained in this work being 2.4% (FWHM = 5.6%). The contribution to variance from photo statistics within the detector material can be estimated as $\frac{2.35}{\sqrt{{\text{N}}_{\text{e}-}}}$ = 0.7%, where N_e_ is the number of photoelectrons collected^16^. Though the intrinsic resolution of the QD bearing GaAs crystals is yet to be fully quantified, device models indicate excitation along steep collection efficiency gradients within the scintillator crystal accounts for most of the remaining variation.

## Conclusion

Nanotechnology has enabled the harnessing of quantum mechanics for the creation of scintillator materials with an unparalleled combination of speed, brightness and energy resolution (Table 1). Successful integration of a spectrally matched In_0.35_GaAs photodetector on GaAs was achieved through use of a transparent AlInGaAs graded buffer layer. The principles governing the integrated device response were identified and used quantitatively in experimental design to reduce variance from scintillator-photodetector coupling geometry. Detector material contributions to energy resolution below 6% FWHM were observed, with room for further reduction through improved minimization or avoidance of collection efficiency gradients.

**Table 1:** Properties of high-performance scintillators.

Scintillator	Decay time (ns)	Yield (photons/MeV)	α-Particle σ/E (%)
BaF_2_	0.6	1500	5.5
GAGG:Ce	50	48,700	4.9
LYSO:Ce	40	29,000	18.9
PbWO_4_	7	200	8.6
QD Scint	0.3	30,000*	2.4

Comparison of properties for some common high-performance scintillators versus our work with epitaxial QD scintillators^18–22^. The fastest component of emission is used for decay time. *The effective QD scintillator yield is given which has PD quantum efficiency as a factor.

The overall efficiency of the devices fabricated to date has been 11% or less and significant potential exists for improvement in light generation and collection. This would positively impact energy resolution and other metrics dependent upon yield such as timing resolution. The fabrication of larger scintillator crystal volumes is crucial for research into sensitivity to neutrons, minimally ionizing particles, gamma rays and the contributions to resolution from proportionality. High energy photons present a unique detection circumstance as these particles create photoelectrons with great range. Under certain conditions these electrons will generate many carriers within the scintillator and photodetector portions of the crystal, resulting in a hybrid detection process.

## Methods

### Alpha particle measurements and response readout

Readout circuits used for alpha particle measurements were designed around Cremat 110 charge sensitive (CS) and 200-1 μs shaping amplifiers (SI Fig. S9). Noise at the output for this readout circuit was measured to be 2188 electrons RMS (SI Fig. S11) vs 2161 based on the components used in the circuit. The Am-241 source with an area of about 2.4 mm in diameter and an activity of 1 μCi has a safety coating that reduced alpha particle energy to 4.4 MeV. The low sigma 5.5 MeV alpha particle uncoated reference source is from the New England Nuclear Corporation with a diameter of 25.4 mm. The sources were characterized in vacuum with an Ortec/Ametek BU-012-100-300 detector (SI Fig. S12 and S13). The resolution of the detector is 12 keV FWHM and was subtracted from the source resolution values given in Fig. 4c. Trigger levels just above the noise (> 3σ_Noise_) were used and data was captured with a 4 GHz Keysight 9404A oscilloscope. The oscilloscope was configured to use a 1 MΩ input impedance along with a 20 MHz low pass filter and simple leading edge trigger.

### Device characterization

TEM data was collected with a FEI Titan 80–300. I–V data was collected before wire bonding with a Keithley 4200. AFM and SEM data was collected with Digital Instruments Dimensions 3100 and LEO 1550 systems, respectively. A 510 nm laser was used for excitation scan data which was collected with the Keithley 4200. Photoluminescence excited by a 50 mW 650 nm unfocused laser was dispersed with a 0.5 m monochromator and collected using a p-i-n InGaAs photodiode with a lock-in amplifier.

### Analytical device model

A 2D response function model is used. The photon-centric spherical expression^5^ is replaced with the crystal centric cartesian expression Eq. (1), as shown in the SI Sect. 3. Alpha particles deeply penetrate the 20–25 μm thick scintillators, so an averaged position mid-thickness was used. Excel was used to calculate the position dependent R = 0,1 and > 1 plane angles for a single PD device, including escape losses. To derive propagation distance values for attenuation, intermediate plane angle positions were used. R > 1 vs. R = 0,1 plane angles are calculated separately and the greater of the two groupings is used to determine the overall device response for excitation at that position.

### Processing

Material growth in terms of layer thickness, composition and deposition temperature are given in Fig. S1. Processing has been previously described^4–6^. Metalized and cleaved specimens are adhered to a substrate by Nagase ChemteX dry film resist DF-1005 under heat. Once placed, the crystal electrodes are wire bonded to the readout circuit with a West Bond 7400A wedge bonder.

### Simulation

The Monte Carlo application was created within MATLAB and consists of a ray tracing loop within a 3-dimensional virtual environment. The application uses relevant physical parameters (indices of refraction, device dimensions, incident particle characteristics, etc.). Alpha particle range is simulated and photons to be ray-traced are randomly generated along this vector. Total internal reflection from surfaces with fixed scattering coefficient is employed for interface interactions. Photon termination methods such as detection, absorption, scattering, and escape are tabulate (see SI Section 2).

## Supplementary Information

Supplementary Information.

## Data Availability

Original characterization and device response data in support of this work has been placed in the online repository Open Science Framework, located at https://osf.io/5ebsk/?view_only = d56a4e0af5594d00b79034db34fecdcd.
