# Supplementary material for: Device response principles and the impact on energy resolution of epitaxial quantum dot scintillators with monolithic photodetector integration
Source: Sci Rep. 2024 Oct 2;14:22870. doi: 10.1038/s41598-024-74160-7 (PMC11445416; doi:10.1038/s41598-024-74160-7)
Supplement: Supplementary file 1 — Supplementary Information. [file 41598_2024_74160_MOESM1_ESM.pdf]

Device response principles and the impact on energy resolution of epitaxial quantum dot scintillator with monolithic photodetector integration

Supplementary information

Contents

Supplementary section 1: Information on crystal growth 2
Supplementary section 2: Monte Carlo simulation 3
Supplementary section 3: Analytical device model 5
Supplementary section 4: Alpha particle measurements and response readout 8

Supplementary section 1: Information on crystal growth

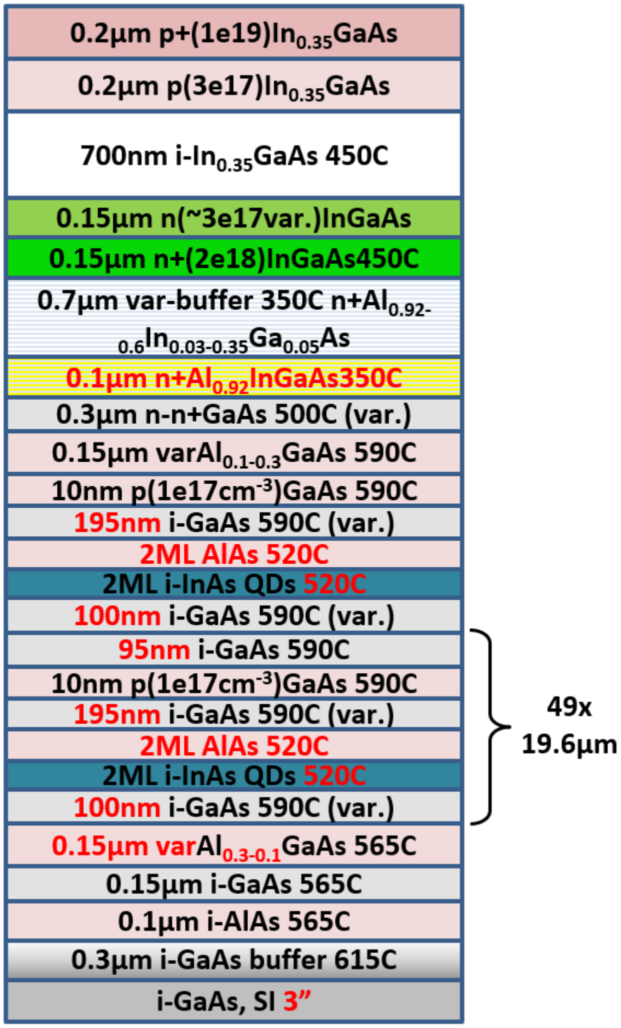

Figure S1: MBE growth parameters by layer.

Supplementary section 2: Monte Carlo simulation

The Monte Carlo application was created using MATLAB and allows large scale raytracing within a virtual device. The application calculates the number of scintillation photons generated along with their trajectories and termination methods. The major components of the algorithm are as follows:

1) Simulation inputs

- Material properties (index of refraction, absorption & scattering coefficients)
- Scintillator & photodetector dimensions
- Overall efficiency
- Number, energy & location of ionizing particles

2) Excitation loop (while n <= number of alpha particles)

Determine penetration vector into scintillator volume at excitation location

Ray trace loop (while i <= number of iterations/photons)

Randomly emit photons isotopically along this vector

Determine ray propagation vector through many reflections

Scattering losses are applied as 1- γ per reflection

Terminate if:

Photon propagates into upper or lower photodetector area

Photon escapes total internal reflection

Photon 'intensity' decays to 1/e due to path length

Surface scattering is applied as a fixed loss per reflection

Append termination location, path length, and method to vector variable

3) Calculate charge collection values, save & print results (Fig. S7 & Table 1)

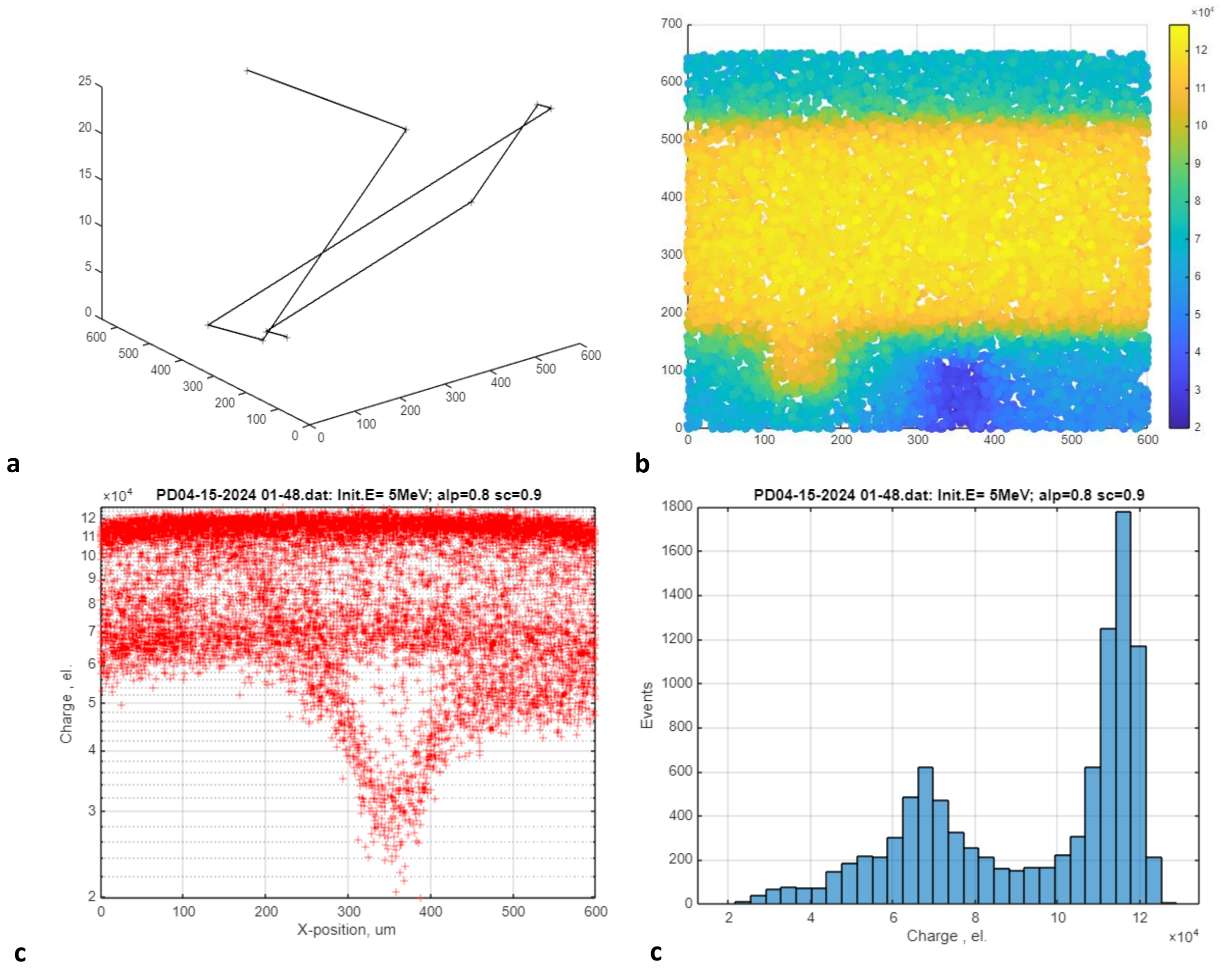

Figure S2: Monte Carlo results modelling the device from Fig. 4 with flood exposure (without aperture substrate). a, 3D ray trace plot of individual photon trajectory which terminated in escape. **b,** 2D Heat map results. **c,** Charge collection along x-axis. **d,** Charge collection histogram. SC is the scattering coefficient, which is equal to 1-γ.

α-particle #	α-particle x (μm)	α-particle y (μm)	Photon #	Path length (μm)	Terminated @ x (μm)	Terminated @ y (μm)	Terminated @ z (μm)	Travel time (ps)	Termination method
1	549	325	1	26	555	347	25	0	Detected
1	549	325	2	21	536	333	25	0	Detected
1	549	325	3	36	551	361	25	0	Detected
1	549	325	4	46	529	285	25	1	Detected
1	549	325	5	28	551	298	25	0	Detected
1	549	325	6	44	538	365	25	1	Detected
1	549	325	7	70	487	347	25	1	Detected
1	549	325	8	706	135	153	25	8	Detected
1	549	325	9	29	540	298	25	0	Detected
1	549	325	10	788	175	348	25	9	Detected
1	549	325	11	29	553	297	25	0	Detected
1	549	325	12	73	513	263	25	1	Detected
1	549	325	13	568	0	467	4	7	Escaped
1	549	325	14	80	536	246	25	1	Detected
...	...	...	...	...	...	...	...	...	...

Table 1: **Tabulated termination method results from the simulation.**

Supplementary section 3: Analytical device model

The response function model approximation used employs Equations 2 and 3 numerically within Excel. Device dimensions, material absorption, device efficiency, particle energy, excitation location and surface scattering coefficients are taken as inputs. The various PD R value plane angles are calculated, normalized over 360 degrees (2π radians) and scaled by the number of photons generated. Attenuation due to photon path length is applied using an average displacement from an averaged position within a given plane angle partition, as shown in figures S3-S6. The same is done when determining the angle of incidence in scattering loss calculations, and an average wavelength of 1100 nm is used. R>1 plane angles are the remainder of 360 degrees – (R=0,1 + escape losses) and their propagation distances forward and backward (reflected) to the PD are calculated. $Z_{photons}$ is an integer number of photons generated by ionizing particle:

$$\mathbf{Z_{photons}} = \frac{E_p F_p \eta_{QD}}{E_{e-h}}$$

$\mathbf{E_p}$ = Ionizing particle energy, $\mathbf{F_p}$ = Fraction of particle energy deposited into scintillator, $\boldsymbol{\eta_{QD}}$ = Quantum dot efficiency, $\mathbf{E_{e-h}}$ = Electron-hole pair creation energy

As stated in the methods section, the R=0,1 partitions are calculated in parallel to the R>1 partitions. As they tend to exist at the expense of each other, the greater of the two groupings is used. Escape wedges are calculated using the index of refraction inputs and excitation location. For device modelling the top escape wedge is removed when excitation happens under the PD. A more precise plane angle analysis was conducted to assess the maximum consumption of escape wedges by PD plane angles.

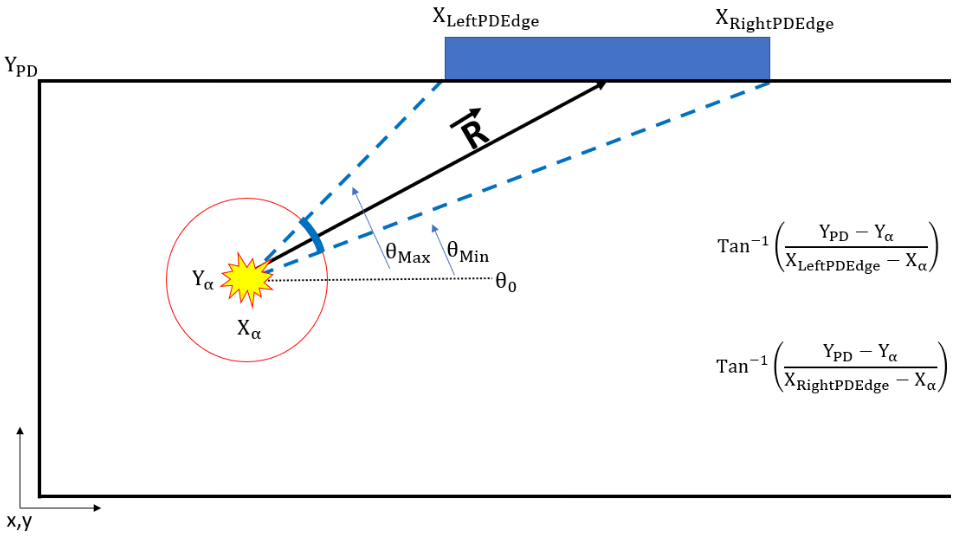

Figure S3: Calculating the R=0 plane angle for an excitation position outside the PD area.

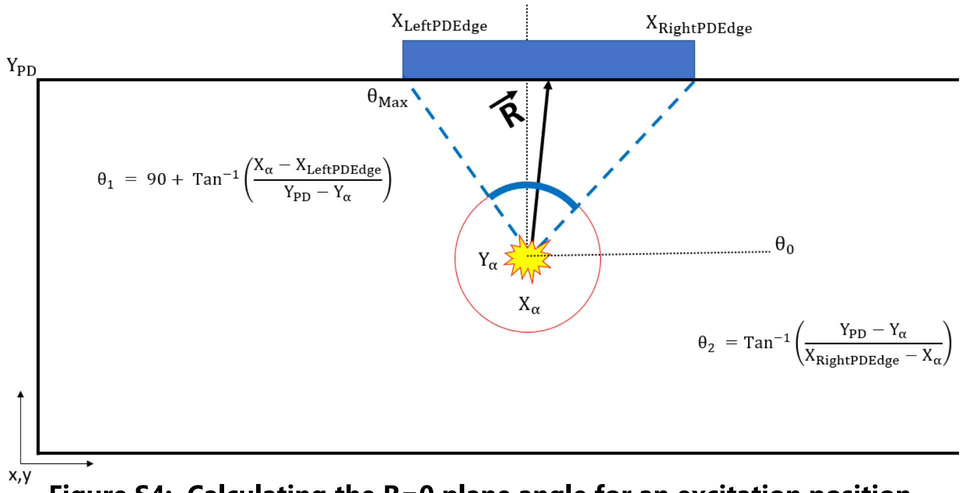

Figure S4: Calculating the R=0 plane angle for an excitation position under the PD.

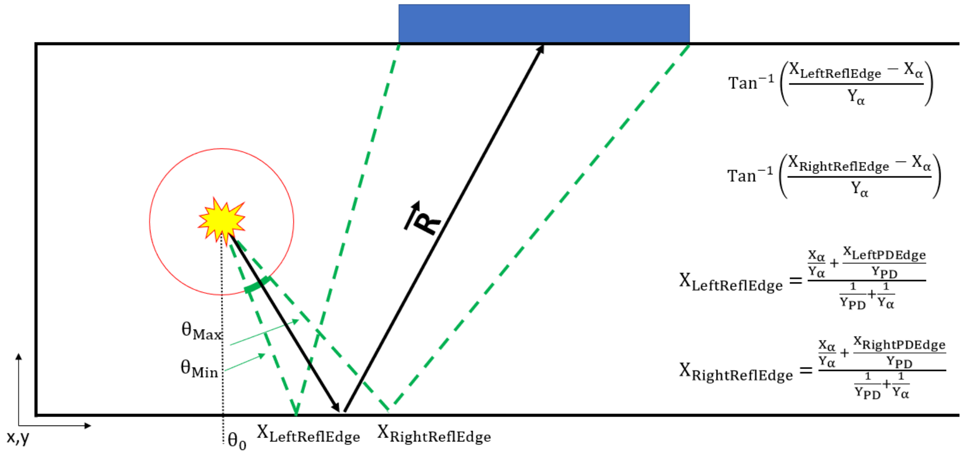

Figure S5: Calculating the R=1 plane angle for an excitation position outside the PD area.

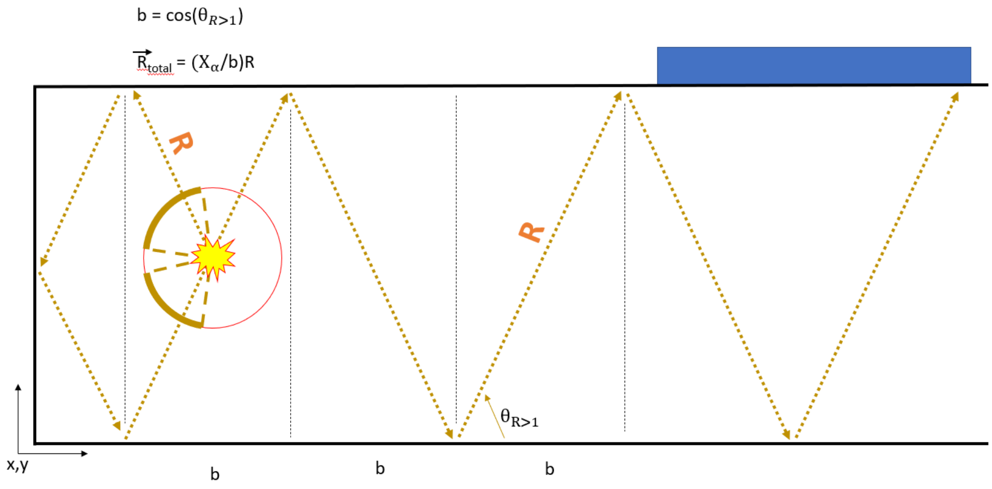

Figure S6: Calculating the averaged forward & backward displacement vectors using a segmented approach. An intermediate angle was used between the escape wedges. The number of reflections for use in the scattering parameter may be derived with b and the emission position.

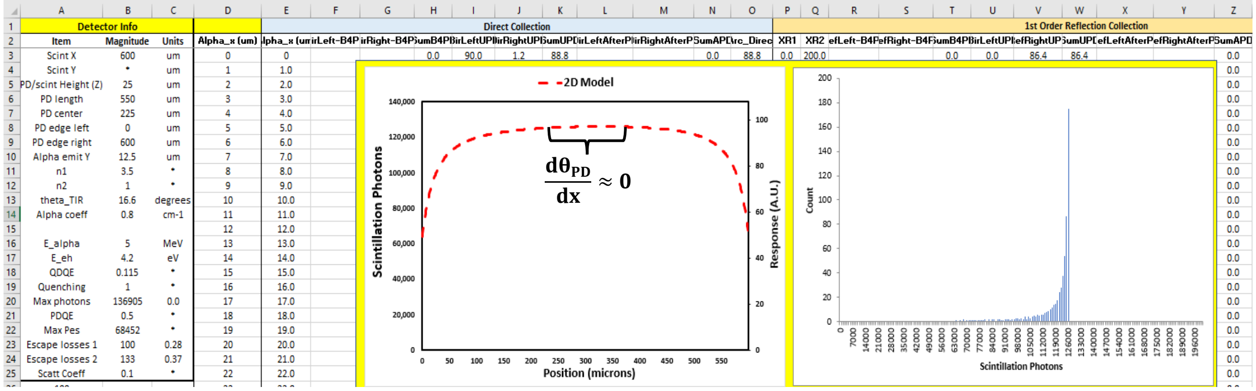

Figure S7: Iterative use of the response function model in Excel to quantify the dimensions of the region of minimal variance. This device geometry presents a region of minimal variation 169 microns wide, centered under the photodetector.

Supplementary section 4: Alpha particle measurements and response readout

The partially optimized readout circuits were designed and built internally and based around commercial components as noted in the methods section. The aperture substrate device was mounted on a small portion of PCB with a hole drilled through, as shown in Fig. S8. The parasitic capacitance from this hardware is significantly less than the PD capacitance. Noise of the CS readout circuit was characterized by acquiring 1 ms samples of background noise for analysis in MATLAB. A Fourier transform is taken to observe the noise spectrum, and the √variance is calculated. Here we find $\mu_{noise} \approx 0$, $\therefore \sigma_{noise} \approx V_{RMS}$. Estimations of the variance from the experimental geometry (source-detector separation in air) were made by hand and with Geant4. For charge sensitive readout, the amount of charge collected is found using the shaping amplifier gain and output pulse amplitude, charge sensitive amplifier gain, and voltage division (which in this case is negligible).

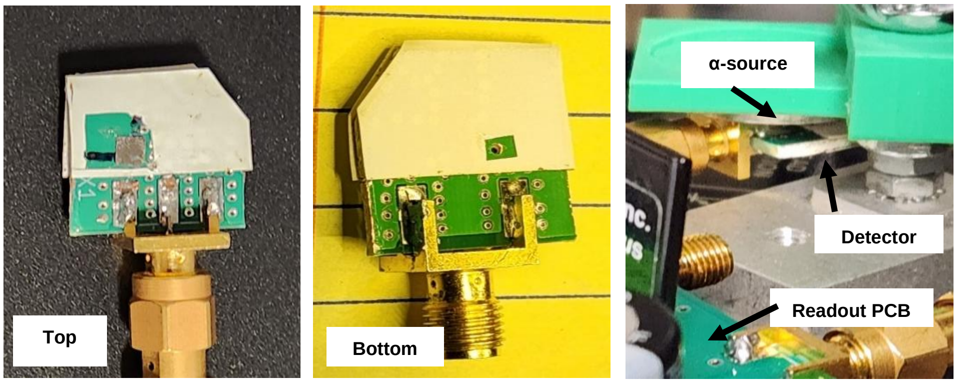

Figure S8: Low magnification images of the detector device under test from Figure 4 along with the experimental configuration.

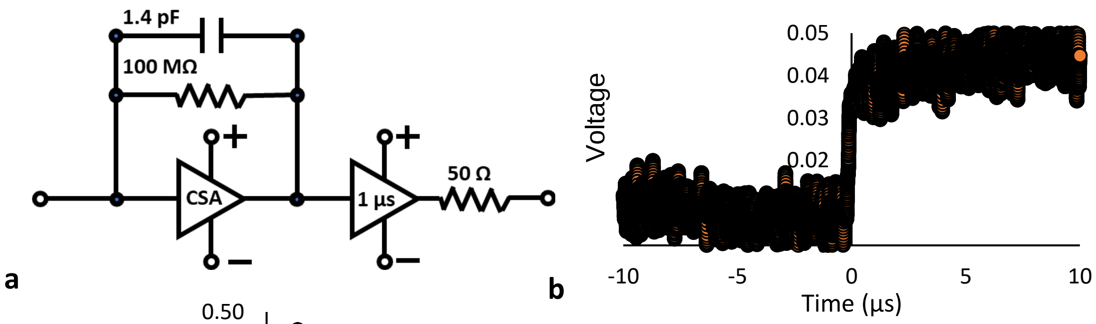

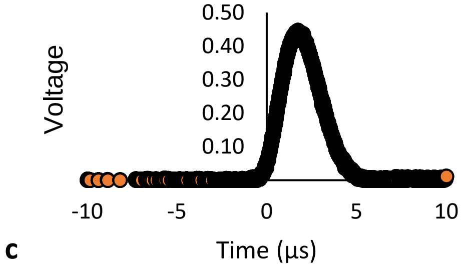

Figure S9: a, Simple diagram of the readout circuit used. The CSA stage is followed by a 10x voltage gain stage with a 1 μs shaping time. **b&c,** Alpha particle responses taken from the CSA and shaping outputs respectively.

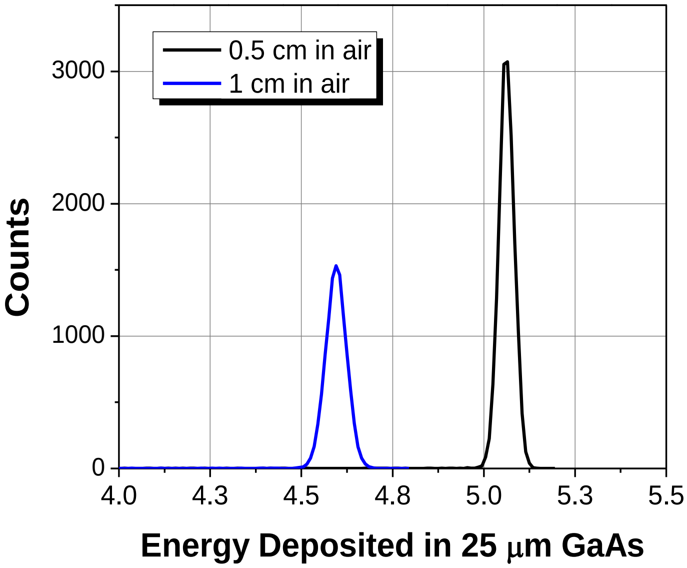

Distance in air	Expected widening (σ/μ)
0.5 cm	0.413%
1 cm	0.62%

Figure S10: Estimating the variance from source-detector separation in air with Geant4. A manual analysis produced results in agreement which were used in Fig. 4.

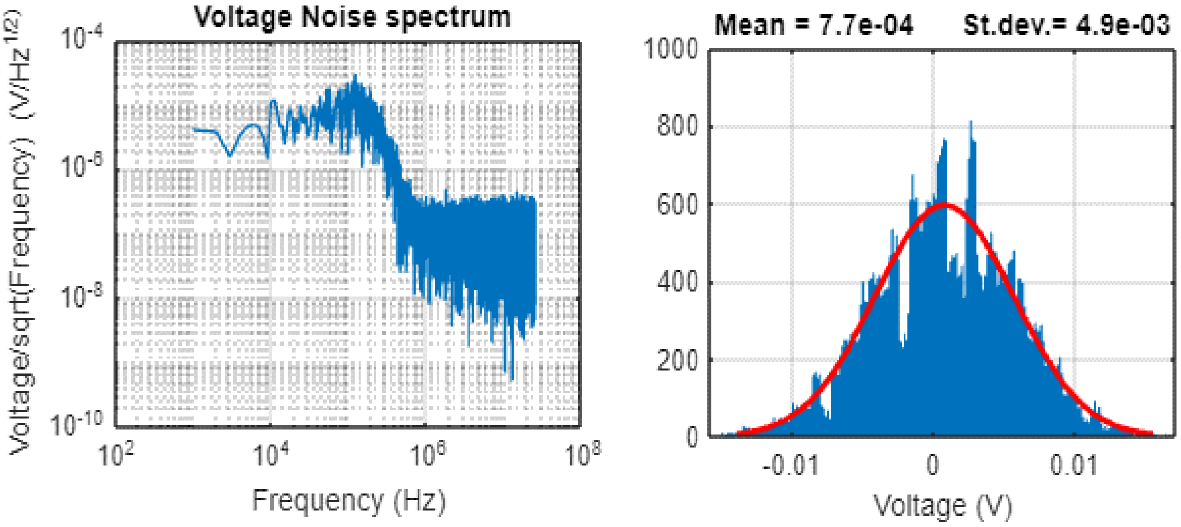

Figure S11: 1 ms noise sample of a charge sensitive readout device without a detector attached. Left, Fourier transform of a waveform reveals the noise spectrum. **Right,** The mean voltage noise is effectively zero volts, RMS voltage ≈ voltage standard deviation. A σ of 4.9e-3 translates to 2,188 electrons RMS for the CS readout circuit used.

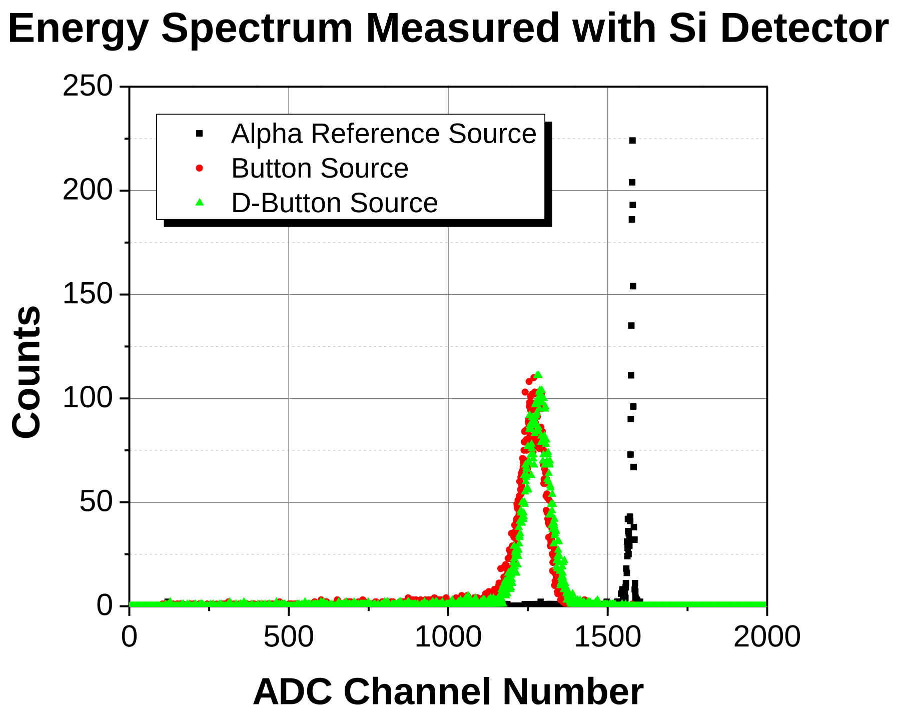

Figure S12: Energy spectrum of the 4.4 MeV and 5.5 MeV sources.

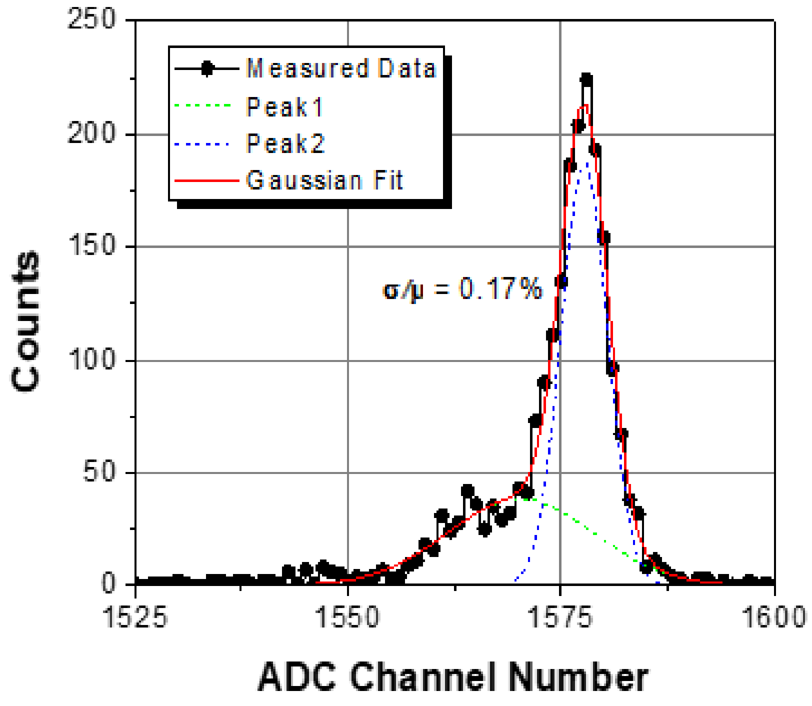

Figure S13: A closer look at the 5.5 MeV uncoated alpha source.
